# COVID-19 in Libya: Fewer Cases So Far. Any Speculations?

**DOI:** 10.1017/dmp.2020.177

**Published:** 2020-05-29

**Authors:** Qais Gasibat, Ali Ahmed Raba, Anis Abobaker

**Affiliations:** Faculty of Medical Technology, National Board for Technical and Vocational Education, Misurata, Libya; School of Medicine, Misurata University, Misurata, Libya; Spire Fylde Coast Hospital, Blackpool, UK

The novel coronavirus disease 2019 (COVID-19) was first reported in December 2019 in Wuhan, China. Since then, the infection has spread worldwide, and it was declared as a global pandemic by the World Health Organization (WHO) on March 11, 2020.^1^ Public health measures have been implemented to combat the spread of the infection and to protect those who are the most vulnerable individuals in communities. The COVID-19 outbreak has been a massive challenge to almost every country across the globe. According to the WHO, many countries are not very well prepared to fight the pandemic. Libya, for example, is more vulnerable to the impact of this pandemic, given the fact that there is an ongoing conflict among the rival factions seeking control of Libya. Libyan citizens have been victims of armed conflicts, with most of the people being subjected to direct and indirect negative consequences. As a result of this conflict in Libya, its economy, health care services, water, and food supply have been hugely affected. For the above-mentioned reasons, Libya is at high risk if the spread of COVID-19 increases. Therefore, the importance of implementing proactive preventive measures to limit the spread of the infection must not be underemphasized.

On March 24, 2020, the first confirmed case of COVID-19 in Libya was reported. According to the National Center for Disease Control in Libya, case zero was an elderly man who returned to the country from a foreign country on March 4, 2020, and he developed symptoms almost 2 weeks later. Since then, the number of cases has increased steadily, and, by April 16, the number of confirmed COVID-19 cases in Libya was 49. This is considered a relatively small number, compared with countries in the region and in the world. So far, only 1 death has been reported of a woman who was tested positive for COVID-19.

There are various potential reasons behind Libya having a small number of COVID-19 cases. First, the government has implemented a series of aggressive measures, even before any case was reported, that seem to be effective in controlling the spread of the virus. Among the control strategies applied include a partial lockdown; the closing of airports, mosques, educational institutions, and crossing borders; and imposing a ban on mass gatherings, as well as limiting movements between the cities. Second, Libya has few airports that connect the country with a small number of foreign countries; therefore, this could be a major reason behind the number of imported cases of COVID-19 being very small. Third, in Libya, the Bacillus-Calmette-Guerin (BCG) coverage is very high, at 98%.^2^ Interestingly, countries with a national program of whole population BCG vaccination appeared to have a lower incidence and death rate from COVID-19.^3^ This may be due to the known immunological benefits of BCG vaccination, which has the possibility to provide a cross protection against severe acute respiratory syndrome coronavirus 2 (SARS-CoV-2), the virus that causes COVID-19. The weather could be another factor that has kept Libya safe from having a large number of cases. Air temperature might play a role in spreading respiratory viral infections.^4^ For instance, cold weather tends to increase the risk of infections by making the respiratory system sensitive. Another important factor behind the small number of confirmed cases in Libya could be the probability of having a limited number of COVID-19 testing kits. According to the National Center for Disease Control, only 634 tests were conducted. Despite having limited test facilities, there was no unexpected rise in the number of suspected patient cases admitted to intensive care units. Furthermore, the social nature of Libya makes it difficult for citizens to visit doctors even if some of the symptoms of COVID-19 are observed. There is a social stigma in which it becomes embarrassing and shameful if anyone has even mild symptoms or has been in contact with someone who tested positive for COVID-19. Therefore, in most cases, citizens avoid being tested. However, the few cases reported in Libya should not be a reason for the country to be adamant about getting prepared for the worst. While Libya has a reason to be relieved for having a small number of COVID-19 cases, it has to consider the fact that it is vulnerable and potentially no more resilient to SARS-CoV-2.
